# Suspended

**Published:** 1884-01

**Authors:** 


### Suspended.

And it is to be hoped only suspended, and that for a short time.

In the December No. of the *Missouri Dental Journal* we regret to find the following:

"This number of the *Journal* closes the fifteenth consecutive volume of its existence, and is the last that will be issued for the present. It pains us greatly to make this announcement for we have used our best endeavors to give the dental profession of the West a first-class journal, devoted especially to their interests, feeling that such an organ should and would meet with proper encouragement at the hands of those in whose behalf it was laboring." The editor further extends thanks to all who have in anywise contributed to the support of the journal.

This step on the part of the editors and publishers will be a matter of regret to the entire profession of the West.

We have ever looked with interest and pleasure to the monthly coming of the Missouri Journal, for it always contained profitable matter.

The profession of the West should not have permitted its suspension for want of support, and I doubt not, when too late, they will realize that they have made a mistake. This journal has exercised a large influence on the profession in the West, and, indeed, it has ever been regarded as one of our reliable dental journals.

May it ere long arouse from its slumber, refreshed, and all the stronger for its rest.

